# Biocontrol of Fall Armyworm Larvae by Selected Mexican *Metarhizium rileyi* Isolates Under Greenhouse and Small-Scale Field Conditions in Maize

**DOI:** 10.3390/insects16070706

**Published:** 2025-07-09

**Authors:** Yordanys Ramos, Samuel Pineda-Guillermo, Patricia Tamez-Guerra, Javier Francisco Valle-Mora, José Isaac Figueroa-de la Rosa, Selene Ramos-Ortiz, Luis Jesús Palma-Castillo, Ana Mabel Martínez-Castillo

**Affiliations:** 1 Instituto de Investigaciones Agropecuarias y Forestales, Universidad Michoacana de San Nicolás de Hidalgo, Km. 9.5 Carretera Morelia-Zinapécuaro, Tarímbaro 58880, Michoacán, Mexico; yordanys.ramos@umich.mx (Y.R.); jose.figueroa@umich.mx (J.I.F.-d.l.R.); ljpalmac@gmail.com (L.J.P.-C.); 2 Facultad de Ciencias Biológicas, Universidad Autónoma de Nuevo León, Av. Pedro de Alva s/n, Ciudad Universitaria, San Nicolás de los Garza 66455, Nuevo León, Mexico; patamez@hotmail.com; 3 Departamento de Ciencias Básicas, Tecnológico Nacional de México, Km. 2 Carretera a Puerto Madero, Tapachula de Córdova y Ordóñez 30700, Chiapas, Mexico; jav.valle@tapachula.tecnm.mx; 4 Secretaría de Ciencia, Humanidades, Tecnología e Innovación (SECIHTI), Instituto de Investigaciones Agropecuarias y Forestales, Universidad Michoacana de San Nicolás de Hidalgo, Km. 9.5 Carretera Morelia-Zinapécuaro, Tarímbaro 58880, Michoacán, Mexico; selene.ramos@umich.mx

**Keywords:** entomopathogenic fungi, biological control, integrated pest management, natural enemies, phytophagous insects, fungi virulence

## Abstract

The efficacy of two selected *Metarhizium rileyi* Mexican isolates (T9-21 and L8-22) against *Spodoptera frugiperda* was evaluated under greenhouse conditions. To this end, a suspension (1 × 10^8^ conidia/mL) of these isolates was sprayed on maize plants previously infested with six second-instar larvae. No significant differences were observed between the survival curves of the T9-21 and L8-22 isolates. Cadaver sporulation was significantly higher, and the lethal time was significantly lower with the T9-21 isolate compared with those of the L8-22 isolate (97% and 8 days vs. 70% and 10 days, respectively). Based on these results, a small-scale field trial on maize was performed to evaluate the degree of pest control achieved by the T9-21 isolate and compare it with the insecticide spinetoram, applied at a rate of 1 × 10^13^ conidia/ha and 75 mL/ha, respectively. No significant differences were observed in the proportion of larval mortality between the T9-21 isolate (0.49) and spinetoram (0.72). However, spinetoram significantly reduced natural enemies and phytophagous insect populations compared with the fungus and the control. In conclusion, *M. rileyi* T9-21 isolate could be a promising alternative for the control of *S. frugiperda* larvae.

## Simple Summary

The fall armyworm (*Spodoptera frugiperda*) is a destructive pest that can severely reduce maize yields around the world. In this study, we tested two Mexican isolates of the entomopathogenic fungus *Metarhizium rileyi* (T9-21 and L8-22) to evaluate their potential for controlling this pest. Greenhouse experiments showed that both isolates caused similar levels of larval mortality, but isolate T9-21 killed the larvae faster and produced a greater proportion of cadavers presenting sporulation. In field trials, isolate T9-21 was as effective as the insecticide spinetoram in killing larvae. However, spinetoram reduced the populations of beneficial insects and other plant-feeding species. These results suggest that *M. rileyi* T9-21 isolate is a promising biological control agent for managing *S. frugiperda* in maize crops and could be safely used in integrated pest management programs.

## 1. Introduction

The fall armyworm *Spodoptera frugiperda* (J. E. Smith) (Lepidoptera: Noctuidae) is native to the tropical and subtropical regions of the Americas and is considered the most serious pest of maize (*Zea mays* L.) worldwide [1]. This insect prefers to feed on Poaceae species. However, due to its highly polyphagous behavior, it may also feed on more than 350 species belonging to 42 plant families [2]. In maize crops, *S. frugiperda* larvae feed on leaf whorls, tassels, and cobs, causing losses of up to 60% of this cereal grain [3]. Moreover, damage caused by these larvae is proportionally higher with increasing larva instars [4].

Globally, chemical control through the use of neonicotinoid, organophosphate, carbamates pyrethroid, spinosyn, and avermectin insecticides is the most practical method for *S. frugiperda* management in the field [3]. However, it has a limited impact because of the insect’s potential to develop resistance [5]. Other factors include the decline of natural enemies’ populations in frequently chemically treated crops and outbreaks of secondary pests [6,7]. Effective and environmentally safe alternatives for *S. frugiperda* control are thus needed for inclusion in integrated pest management (IPM) programs. One promising control strategy against these pest larvae is biological control with entomopathogenic fungi, which are highly reliable since they have been successfully implemented against many agricultural pest species [8].

Although entomopathogenic fungi depend on environmental conditions for the successful completion of their infective cycle and typically require more time to kill insect pests, one of their most notable advantages is their natural occurrence in the environment [9]. This characteristic confers a specific affinity for targeting pests while minimizing impacts on non-target organisms and enables these fungi to adapt and co-evolve with host populations. Consequently, the risk of pests developing resistance is reduced [10]. Unlike other microorganisms used in biological control, entomopathogenic fungi do not need to be ingested, as they act through contact. They can overwinter in the soil and infect the new generation of insect hosts [11]. Additionally, they have the ability to establish themselves as endophytes within plants, providing protection against herbivory from insect pests [7]. Moreover, their potential to negatively affect the environment, crops, or human health is minimal, making them a sustainable and ecologically sound alternative for pest management [12].

The entomopathogenic fungus *Metarhizium rileyi* (Farlow) Kepler S.A. Rehner & Humber (Ascomycetes: Hypocreales) was previously described as *Botrytis rileyi* (Farlow), *Spicaria rileyi* (Farlow) Charles, and *Nomuraea rileyi* (Farlow) Samson [13]. However, morphological and phylogenetic studies conducted by Kepler et al. [14] placed it within the *Metarhizium* genus. *Metarhizium rileyi* is widely distributed and, like other *Metarhizium* species, is a common inhabitant of the soil [15]. In addition, its host range is restricted mainly to lepidopterans [16]. A suitable temperature of around 25 °C and relative humidity (RH) levels above 75% are critical environmental conditions needed to promote its viability and complete the infection cycle [17,18]. The infection process of this entomopathogenic fungus begins when its conidia come into contact with the host insect’s cuticle; it secretes mucilaginous substances and proteins that facilitate attachment [19]. Once *M. rileyi* enters the larval hemocoel, its cells transform into blastospores, facilitating multiplication within the *S. frugiperda* larva. Finally, after the larva dies, mycelial proliferation occurs through all natural openings of the larva, followed by the formation of conidia [20].

By identifying 24 isolates, our group previously reported that *M. rileyi* is highly pathogenic and virulent against *S. frugiperda* under laboratory conditions, causing high mortality (70% to 99%) in second-instar larvae at 13-days post-inoculation [21]. Other isolates of this fungus also caused similar mortality (70% to 98%) in different instars of *S. frugiperda* when treated with different inoculation methods at a concentration of 1 × 10^8^ conidia/mL [22]. Furthermore, other studies have shown the effectiveness of this fungus against related and lepidopteran species, such as *Spodoptera exigua* Hübner [23], *Spodoptera litura* Fabricius [24,25], *Helicoverpa armigera* Hübner [13], and *Helicoverpa zea* Boddie [26]. Moreover, *M. rileyi* can infect several coleopterans, including *Hypera punctata* (Fabricius), *Leptinotarsa decemlineata* (Say), and *Popillia japonica* Newmane [22]. However, the response of these insects to infection caused by entomopathogenic fungus may vary under uncontrolled conditions. Studies conducted under greenhouse and field conditions have demonstrated that *M. rileyi* infection and virulence in *S. frugiperda* larvae remain steady, even under different climatic factors [27,28,29]. Furthermore, native isolates of entomopathogenic fungi may exhibit distinct biological activity compared to commercial strains, depending on their geographic origin and environmental adaptation. For this reason, the selection of an isolate as a biological control agent requires that native isolates be tested against an insect population from the area in which the program is to be run [30].

The present study aims to evaluate (1) the efficacy of two native isolates of *M. rileyi* against *S. frugiperda* larvae under greenhouse conditions and (2) the impact of the most effective isolate of this fungus on larvae of this pest, as well as on other phytophagous insects and natural enemies, under field conditions.

## 2. Materials and Methods

Conidia germination, microcultures, polysporic, and monosporic cultures, as well as larval incubation processes, were conducted at 25 °C ± 2 °C and 75% ± 5% RH in complete darkness unless otherwise specified.

### 2.1. Insect Rearing

Insect specimens were obtained from a population of *S. frugiperda* maintained at the Entomology Laboratory of the Instituto de Investigaciones Agropecuarias y Forestales (IIAF), Universidad Michoacana de San Nicolás de Hidalgo (UMSNH), Mexico. Larvae were individually reared on an artificial diet without formaldehyde [31] in 30 mL plastic cups. Adults were placed in brown paper bags (18 cm × 11 cm × 40 cm) and fed with a 15% honey solution. Paper bags were replaced daily, following the onset of egg laying. The colony was maintained in an environmental chamber at 25 °C ± 2 °C and 65% ± 5% RH and a 16 h:8 h photoperiod (light–darkness).

### 2.2. Fungal Isolates

*Metarhizium rileyi* T9-21 and L8-22 isolates were obtained from *S. frugiperda* larvae at El Trébol, Tarímbaro (19°46′12.50″ N, 101°09′17.67″ W), and Lagunillas, Pátzcuaro (19°36′40.55″ N, 101°25′23.7″ W), respectively, in the state of Michoacán, Mexico [21]. These isolates were conserved in glycerol at 4 °C (until the fungal isolates were used for subsequent experiments) in IIAF-UMSNH and molecularly and biologically characterized by Ramos et al. [21].

To reproduce these isolates, polysporic cultures were prepared on MPYA culture medium (maltose 40 g/L, casein peptone 10 g/L, yeast extract 10 g/L, and agar 15 g/L) [21]. We used these isolates only if their conidial germination was ≥95%, which is considered adequate for bioassays [32]. For this, microcultures were produced using 14-day-old conidia from the polysporic cultures, according to the technique described by Ramos et al. [21]. The number of germinated conidia was determined 24 h after incubation, and the percentage of conidial germination was calculated according to Rombach [33] as follows:

% conidial germination = (V/T) × 100

where V is the total number of viable conidia, and T is the total number of viable and non-viable conidia in the sample. Conidia were considered germinated when the length of their germ tube was at least twice the length of the conidia [34].

*Metarhizium rileyi* isolate monosporic cultures were obtained as described by Goettel and Inglis [35], with the exception that conidia were incubated at 25 °C instead of 28 °C. Next, 20-day-old conidia from each monosporic culture were obtained by scraping them into 100 mL of sterile distilled water with 0.05% Tween 80 and mixed for one minute in a vortex. To achieve the desired concentration of isolates for the experiments, conidia counting was performed in a Neubauer chamber (Hausser Scientific, Horsham, PA, USA).

### 2.3. Experiments Under Greenhouse Conditions

Maize seeds (hybrid variety Cb-hs-55, Bayer, Ciudad de México, Mexico) were allowed to pregerminate on a tray lined with a paper towel moistened with sterile distilled water. Seven days later, two pregerminated seeds were sown in polyethylene black bags (15 cm wide × 30 cm high) containing a mixture of humus-rich soil and sand at a ratio of 3 to 1. After a week, the smallest plant was removed to ensure uniform plant size, leaving only one plant per bag.

Maize plants, grown in a greenhouse covered with polyethylene and anti-aphid mesh, were watered three times per week with 150 mL of water adjusted to a neutral pH using a laboratory pH meter (Aquasearcher AB23PH; Ohaus, Parsippany, NJ, USA). Each plant was fertilized once a week with three grams of a granular fertilizer containing nitrogen, phosphorus, and potassium (17-17-17).

Maize plants were grown until reaching the V6 growth stage (~50 cm high), as recommended for this experiment [36]. Maize plants were subjected to one of the following treatments: (i) *M. rileyi* T9-21 isolate, (ii) *M. rileyi* L8-22 isolate, or (iii) water control. Treatments were randomly assigned, and there were five replicates per treatment (one plant per replica) [37]. Both *M. rileyi* isolates were applied at a concentration of 1 × 10^8^ conidia/mL. Previously, plants were infested with six newly molted second-instar *S. frugiperda* larvae, which were placed on their whorl, using a fine brush. These larvae were randomly selected from different batches of insect rearing. After two hours of infestation, plants were sprayed with a suspension of 5 mL per plant of each fungal isolate using a manual sprayer. Each replica was sprayed with a new fungal suspension. To enhance leaf wetting, we used 0.05% Tween 80. Control plants were only sprayed with 0.05% Tween 80 in distilled water. To avoid contamination between treatments, *M. rileyi* T9-21 and L8-22 isolates were applied outdoors, and when dried (two hours after treatment), plants were moved into the greenhouse mentioned above.

After treatment, maize plants were covered with muslin cloth (200 μm mesh) to confine *S. frugiperda* larvae. After two days, plants were transported to the laboratory to recover larvae, which were individually placed in 30 mL plastic cups containing filter paper moistened with sterile distilled water and a ~1 cm^2^ section of semisynthetic diet for feeding. Larvae were then incubated and observed every 24 h until death, and survival was determined. To promote sporulation, dead larvae were individually placed in a 24-well tissue culture plate containing filter paper with 500 µL of sterile distilled water. The proportion of cadavers presenting sporulation was registered. In addition, the median lethal time (LT_50_) was determined using time–mortality data.

### 2.4. Small-Scale Field Trials

We selected the *M. rileyi* T9-21 isolate based on the proportion of *S. frugiperda* larva cadavers presenting sporulation and low LT_50_ obtained in a greenhouse bioassay, as detailed in the results section. This study was conducted in a maize crop, of the variety mentioned above, at the campus of La Posta Zootécnica, Facultad de Medicina Veterinaria y Zootecnia, UMSNH (Tarímbaro, Michoacán, Mexico; 19°46′5.5776″ N, 101°09′1.7028″ W) at 1860 m a.s.l. (https://inegi.org.mx/). The area used for this experiment is exclusively used for maize production, and it is not rotated, intercropped, or adjacent to any other crop.

We established experimental plots of 10 m^2^, spaced 1.30 m apart. Each plot consisted of six rows, spaced 0.8 m apart, containing approximately 33 maize plants. When the maize plants reached the V6 growth stage [36], plots were subjected to one of the following treatments: (1) *M. rileyi* T9-21 isolate at a concentration of 1 × 10^13^ conidia/ha, (2) spinetoram (Palgus^TM^, Corteva, Ciudad de México, Mexico) at the product label-recommended rate of 75 mL/ha), and (3) water control. Spinetoram is a mixture of spynosyn J and L produced during the fermentation of the soil actinomycete *Saccharopolyspora spinosa* [38]. Treatments were randomly assigned to plots using five replicates per treatment. In all experimental plots, each maize plant was infested with three second-instar *S. frugiperda* larvae at 8:00 a.m., avoiding external rows. Next, treatment applications were made with a volume equivalent to 27 mL per plant, most of which was directed into the whorl, using a 15 L manual knapsack sprayer (Model 189030; LoLasafe, Ciudad de México, Mexico), with a full cone nozzle calibrated to release 9 mL of the solution per second. The agricultural wetter–sticker Inex-A (Cosmocel, San Nicolás de los Garza, Nuevo León, Mexico), at a rate of 1 mL/L, was included in all treatments. The application of treatments was conducted at 6.00 p.m. to avoid intense solar radiation exposure and increase the inoculum contact time with the larvae. The percentages of conidia germination, dilution, conidia counting, and concentration adjustment of the *M. rileyi* T9-21 isolate, as well as the recorded water pH data, were performed as described above. Before treatment application (0 days), as well as 2-, 4-, and 6-days post-application, 10 plants per plot were randomly selected and carefully examined. The number of *S. frugiperda* larvae and other arthropods (phytophagous and natural enemies) were then recorded on each plant. The larvae present on each maize plant in all sampling times, with the exception of 0 days, were recovered, placed in 30 mL plastic cups, and transported to the laboratory. Sampled plants were marked to avoid re-sampling in future observations.

In the laboratory, recovered larvae were fed with a semisynthetic diet and incubated in a climate chamber. To promote the proliferation of fungi, filter paper moistened with distilled water was placed at the bottom of the plastic cups. Larvae were observed every 24 h to determine larval mortality. Temperature and RH were collected using a Hobo data logger (UX100-003; Onset Computer Corp., Pocasset, MA, USA), which was placed within the maize plants on a 30 cm height wooden box.

### 2.5. Statistical Analysis

The Gehan–Breslow and Kaplan–Meier survival analyses and the non-parametric LIFETEST procedure were used to compare the effects of two fungal isolates on the survival of *S. frugiperda* larvae in the greenhouse experiment. A pairwise multi-comparison procedure (Long-Rank test, *p* < 0.05) was used to detect significant differences between treatments. To compare the proportion of cadavers presenting sporulation between the fungal isolates, a generalized linear model (PROC GLM) with a binomial distribution was used. The LSMEANS test (*p* < 0.05) was used for means separation (SAS/STAT^®^, version 8.1; SAS Institute, Gary, NC, USA). LT_50_ values were calculated using the Polo Plus software version 1.0 (2002–2003, LeOra Software, Berkeley, CA, USA), and χ^2^ goodness-of-fit was performed for each isolate. Differences in LT_50_ values between isolates were determined based on the non-overlap of 95% Fiducial Limits (FLs).

For the field trial, an analysis of deviance was performed, accounting for treatments, plots, and collection times. A GLM with a binomial distribution was then applied, and Tukey’s HSD test was used for means separation. A canonical biplot analysis was performed to determine the differences in the proportions of natural enemies and phytophagous insects by treatment, followed by a MANOVA test. In addition, two-way ANOVA was performed to analyze the mean number of natural enemies and phytophagous insects. The factors ‘treatment’ and ‘collection time’ were included in each design to test the significant effects of individual factors and their possible interactions. Tukey’s HSD test was used for comparisons of treatment or factor means. All analyses in the small-scale field experiment were performed using R version 4.4.2 (www.r-project.org, accessed on 12 December 2024), setting a significance level of 0.05.

## 3. Results

### 3.1. Greenhouse Bioassay

**Figure 1:**
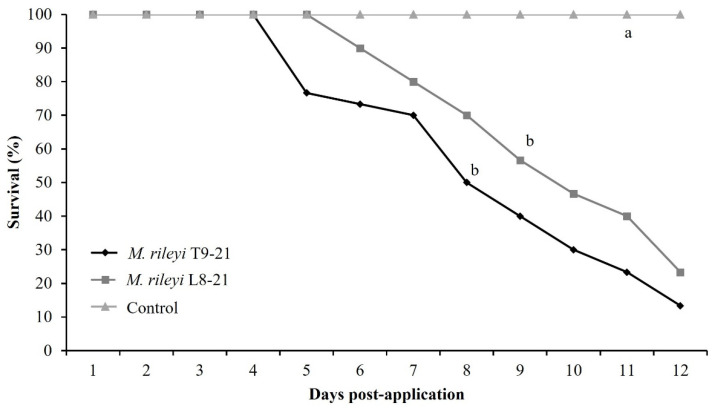
Gehan–Breslow and Kaplan–Meier survival curves for second-instar larvae of *S. frugiperda* after application with a concentration of 1 × 10^8^ conidia/mL of two isolates of *M. rileyi*, under greenhouse conditions. Different letters indicate significant differences according to log-rank test (*p* < 0.05).

Spraying of 1 × 10^8^ conidia/mL of *M. rileyi* T9-21 and L8-22 isolates on maize plants, artificially infested with fall armyworm second-instar larvae, resulted in 13.33% and 23.33% larval survival at 12-days post-application, respectively. No significant differences were observed between the survival curves of these two isolates, but both were significantly different compared with the control (log-rank test, χ^2^ = 50.23, *p* < 0.0001; Figure 1).

The proportion of cadavers presenting sporulation after the isolate T9-21 treatment was significantly higher (97% ± 0.05%, F = 67.61, *p* < 0.001) than that of the L8-22 isolate treatment (70% ± 0.06%). LT_50_ value of the T9-21 isolate was significantly lower (8.17 days, FL_95_ = 7.50–8.50) than that of the L8-22 isolate (9.79 days, FL_95_ = 9.20–10.50). The control treatment did not cause larval mortality.

### 3.2. Small-Scale Field Trial

**Figure 2:**
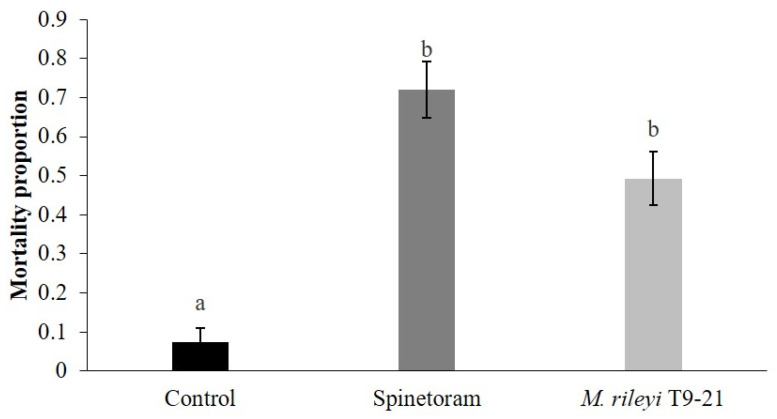
Mortality proportions induced by *M. rileyi* T9-21 isolate, spinetoram, and the control treatment in *S. frugiperda* larvae in maize plants under field conditions. Data represents probability of mortality ± SEM. Different letters indicate significant differences according to Tukey’s HSD test (*p* < 0.05).

Larval mortality did not change over time (days post-application) (χ^2^ = 0.39, *p* = 0.98). The total mortality proportion caused by spinetoram (0.72 ± 0.07) and *M. rileyi* T9-21 isolate (0.49 ± 0.068) was significantly higher (χ^2^ = 46.77, *p* < 0.0001) than that of the control (0.07 ± 0.03; Figure 2).

**Figure 3:**
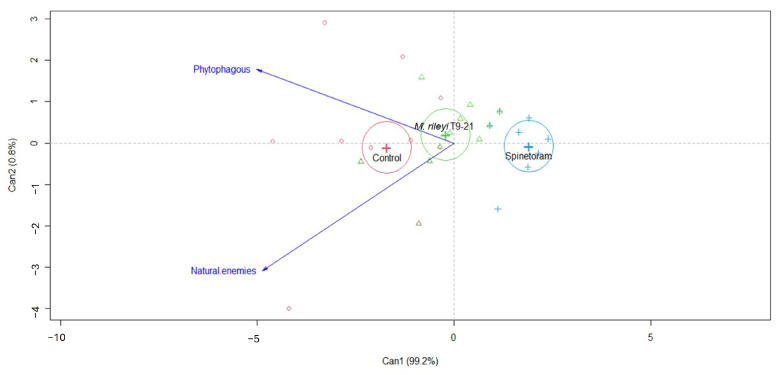
Effect of *M. rileyi* T9-21 isolate and spinetoram application on the abundance of phytophagous insects and natural enemies in maize plants under field conditions.

The canonical biplot analysis showed that the presence of natural enemies and phytophagous insects was significantly less abundant in spinetoram and *M. rileyi* T9-21 treatments compared with the control, regardless of the collection time (F = 10.95, *p* < 0.00001, Figure 3). The most commonly observed natural enemy was the earwig *Doru taeniatum* (Dohrn) (Dermaptera: Forficulidae), accounting for 71.6% of the total natural enemies recorded. Other natural enemies observed included *Orius* sp. (Hemiptera: Anthocoridae), *Collops* sp. (Coleoptera: Melyridae), coccinellids, and spiders. The phytophagous insects recorded were *Diabrotica virgifera* zeae Krysan & Smith, *Diabrotica balteata Leconte* (Coleoptera: Chrysomelidae), *Euschistus heros* Fabricius (Hemiptera: Pentatomidae), and aphids.

**Figure 4:**
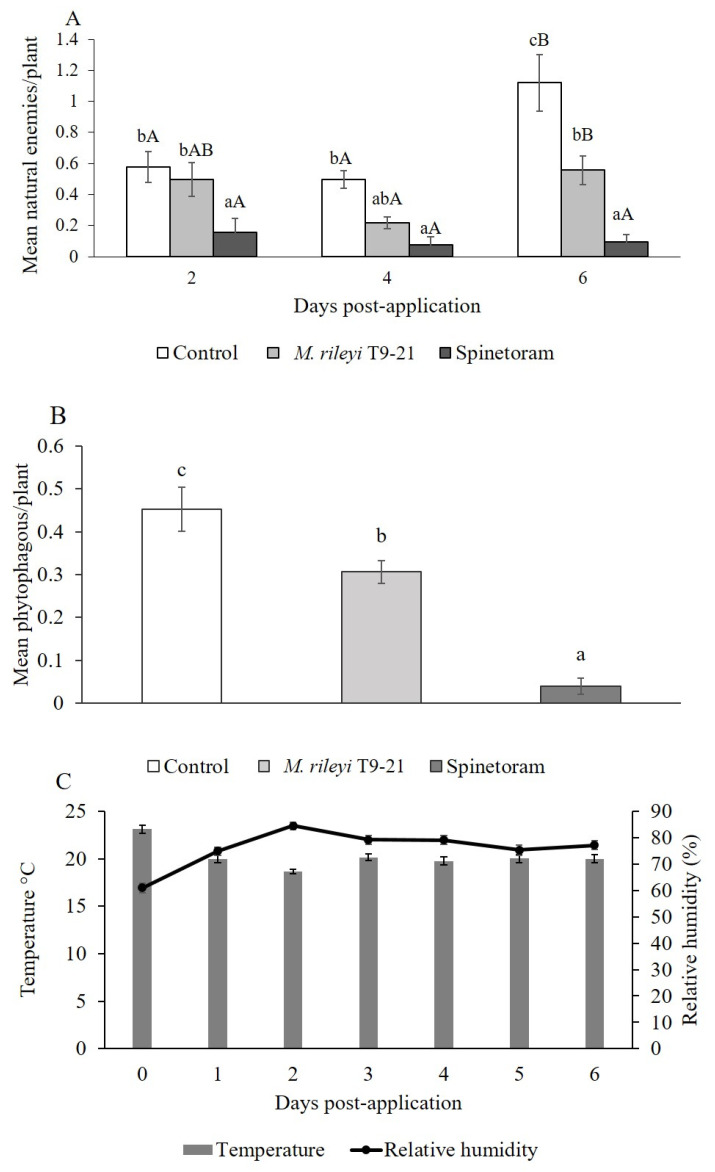
Mean numbers of arthropod natural enemies and phytophagous insects observed in maize plants after application of *M. rileyi* T9-21 isolate and spinetoram, under field conditions. (**A**) Mean number of natural enemies per plant at 2-days, 4-days, and 6-days post-application; (**B**) mean number of phytophagous insects per plant within each treatment; and (**C**) temperature fluctuation and relative humidity at the time at which the treatments were applied (day 0) and 1-day to 6-days post-application. Bars labeled with lowercase letters indicate significant differences between treatments at the same time. Bars labeled with capital letters indicate significant differences between days post-application for the same treatment.

Regarding natural enemies, no significant differences were observed between plots and recorded insects (*p* = 0.51). However, there were significant differences in the factors ‘treatment’ (F = 31.91, *p* < 0.0001) and ‘collection time’ (F = 8.89, *p* < 0.0008). A significant interaction was observed between both factors (F = 3.76, *p* < 0.0124). After application, the natural enemy density was significantly lower with the spinetoram treatment compared with that of the *M. rileyi* T9-21 isolate and control treatments, with one exception (Figure 4A). At 4-days post-application, no significant differences were observed between the *M. rileyi* T9-21 isolate and spinetoram treatments (0.21 ± 0.03 and 0.07 ± 0.04, respectively). Furthermore, no significant differences were observed between *M. rileyi* T9-21 isolate at 2- and 4-days post-application (0.50 ± 0.1 and 0.22 ± 0.03, respectively) and the control (0.58 ± 0.09 and 0.50 ± 0.05, respectively; Figure 4A). However, at 6-days post-application, the natural enemy density was significantly higher than that of the control treatment (1.12 ± 0.18) compared with that of the *M. rileyi* T9-21 isolate (0.56 ± 0.09). The mean of the natural enemies significantly increased 6-days post-application with the *M. rileyi* T9-21 isolate and control treatments compared with previous days, whereas with the spinetoram treatment, recorded data were similar across the three collection times (Figure 4A).

The density of phytophagous insects per plant was only influenced by the ‘treatment’ factor (F = 32.24, *p* < 0.0001). The highest density was observed with the control (0.45 ± 0.05), followed by *M. rileyi* T9-21 isolate (0.30 ± 0.0) and spinetoram (0.04 ± 0.02; Figure 4B).

In this study, temperature and RH ranged from 19 °C to 21 °C and from 70% to 84.4%, respectively. A slight decrease in temperature and an increase in RH were observed two-days post-application (Figure 4C).

## 4. Discussion

*Metarhizium rileyi* is a cosmopolitan pathogen with great potential to control *S. frugiperda* larvae and other noctuid pests [37,39]. Most reports assessing this fungus’s effectiveness as a bioinsecticide against this pest have been conducted under laboratory conditions [40,41]. In the present study, we evaluated the virulence of two Mexican *M. rileyi* isolates against *S. frugiperda* larvae under greenhouse conditions, followed by a small-scale maize field trial to evaluate the degree of control achieved by the most effective isolate in the greenhouse experiment based on the speed of larvae killing and the proportion of cadavers presenting sporulation.

We demonstrated that the *M. rileyi* L8-22 and T9-21 isolates were highly effective in controlling *S. frugiperda* larvae under greenhouse conditions; isolate T9-21 was the fastest to kill the larvae of this insect. These results agree with previous reports testing the same monosporic isolates and finding similar virulence against second-instar *S. frugiperda* larvae in laboratory bioassays [27]. The survival rates obtained for the L8-22 (23%) and T9-21 (13%) isolates under greenhouse conditions also agree with other studies, where the same insect species and host plants were used. Concentrations of 4.03 × 10^6^ conidia/mL and 6.3 × 10^6^ conidia/mL of one *M. rileyi* Colombian isolate (Nm06) [27] and one Brazilian (CG381) isolate [28] applied to plants previously infested with second- and third-instar larvae caused a survival rate of 21% and 12% at 11-days and 8-days post-application, respectively. Although a high concentration of isolates (1.0 × 10^8^ conidia/mL) was applied to maize plants in our study compared with previous studies, differences in insect host susceptibility may be related to genetic differences between the *M. rileyi* isolates and/or experimental conditions.

The pathogenicity of *M. rileyi* isolates evaluated in our study was higher than that reported for other entomopathogenic fungi against *S. frugiperda* larvae. Two *Beauveria bassiana* (Balsamo-Crivelli) Vuillemin isolates from Thailand, applied under greenhouse conditions at rates of 1 × 10^8^, 1 × 10^9^, and 1 × 10^10^ conidia/mL on maize plants, caused mortality rates of 19% to 21%, 20% to 25%, and 32% to 35%, respectively, 10-days post-application in second-instar larvae of this insect [42]. Idrees et al. [43] determined that a Chinese *B. bassiana* isolate sprayed on maize leaves at a rate of 1 × 10^8^ conidia/mL caused 16% mortality in second-instar *S. frugiperda* larvae at 7-days post-treatment. These results suggest that *M. rileyi* isolates, used in the present study, are highly pathogenic to *S. frugiperda* and have the potential for use as bioinsecticides. However, differences in biological activity are strongly influenced by the susceptibility of the insect host and probably genetic differences between isolates.

The sporulation of entomopathogenic fungi on cadavers is an essential source for the transmission of spores to susceptible hosts [44]. In our study, the isolate T9-21, which killed *S. frugiperda* larvae in less time, exhibited a higher sporulated cadaver rate (97%) compared with that of the isolate L8-22 (69%). This is consistent with other studies in which a positive correlation between mortality and the proportion of cadavers presenting sporulation among *M. rileyi* isolates was reported [18,21]. Other studies are needed to determine possible differences in the infection process and physiological and biochemical effects between the isolates studied here to identify key factors that can influence the host’s susceptibility.

Our small-plot field experiment demonstrated that applying isolate T9-21 at 1 × 10^13^ conidia/mL caused 50% mortality in *S. frugiperda* larvae across all collection times. In other maize field experiments, Lopes et al. [45] determined that either conventional spraying or drone application of a Brazilian *M. rileyi* isolate (CG381), at a concentration of 5 × 10^12^ conidia/ha, caused 48% and 35% larval mortality in *S. frugiperda*, respectively. Studies with the same Brazilian isolate demonstrated that spray applications at concentrations of 0.6 and 1.2 × 10^12^ conidia/ha or 2 × 10^7^ and 2 × 10^8^ conidia/mL resulted in larval mortalities of 27–31% [28] and 13–38%, respectively [29].

Differences in mortality caused by *M. rileyi* among studies by Faria et al. [22], Barros et al. [29], Lopes et al. [45], and ours may be attributed to fungal concentrations, host susceptibility, and/or the age of the host at the time of fungal application. In addition, the susceptibility of *S. frugiperda* to different *M. rileyi* isolates largely depends on overcoming the defenses induced by the insect pest [46,47] and the presence of suitable environmental conditions needed to conserve its conidial viability and to complete the infective cycle [29,48]. Favorable and stable temperature conditions (19 °C to 21 °C) and RH (70% to 84%) prevailed during our study, which agrees with the optimal values needed to promote the complete infection cycle of *M. rileyi* (temperature 18 °C to 26 °C and RH > 75%) [17,18]. In addition, because entomopathogenic fungi are degraded by UV light [49], we applied treatments in the late afternoon (6:00 p.m.) to avoid intense solar radiation and increase the contact time of conidia with larvae for the remaining day and night.

Although no significant differences were observed in larval mortality between the spinetoram (72%) and *M. rileyi* T9-21 isolate (50%) treatments in this study, the density of natural enemies and other phytophagous insects was significantly lower in the insecticide compared with that of *M. rileyi* and control treatments. These results agree with those reported by Méndez et al. [50], who reported that spinosad (an insecticide from the same family as spinetoram, which is produced through the fermentation of the soil actinomycete *Saccharopolyspora spinosa*) [38] significantly reduced the density of natural enemies and other insects in a maize crop. In addition, these authors determined that this effect may be variable among insect species and positively dose-dependent. In our study, the predators *D. taeniatum* and *Orius* spp. were the most prevalent natural enemies. This is consistent with the findings of Méndez et al. [50] and Cisneros et al. [51], who reported that these predators were also the most abundant and the most sensitive to the applied insecticide, which may also have occurred in this study.

It is widely known that *M. rileyi* exhibits host specificity for larvae of the Noctuidae family, which minimizes the risk to non-target organisms [16,52]. We have observed that the presence of natural enemies and other phytophagous insects tended to decrease in the *M. rileyi* treatment, compared with the control, at 4-days post-application, although the differences were not statistically significant. However, in the last collection time (6-days post-application), a significant difference was observed between these two treatments. We have no clear explanation for this result. However, we believe that this may be due to deterrence effects caused by *M. rileyi*. This statement is supported by the studies by Meyling et al. [53]. They found that the generalist predator *Anthocoris nemorum* L. (Heteroptera: Anthocoridae) detected and avoided contact with *Urtica dioica* L. leaves treated with the entomopathogenic fungus *B. bassiana*. These authors attributed this effect to the detection of fungus-specific cues, apparently associated with compounds present on the surfaces of the conidia. In addition, Myles [54] showed that *Metarhizium anisopliae* (Metchnikoff) Sorokin caused alarm, aggregation, and defensive reactions in termites when exposed to this fungus. However, this response appears to be specific and adaptive [54]. It will be of interest to perform further studies to identify the related factors concerning the possible impact of *M. rileyi* on non-target insects associated with maize crops.

## 5. Conclusions

This study demonstrated that the selected *M. rileyi* Mexican T9-21 and L8-22 isolates were effective in controlling *S. frugiperda* larvae under greenhouse conditions. Due to its comparable effectiveness with spinetoram, the selected and field-tested *M. rileyi* T9-21 isolate may be considered in integrated pest management programs for *S. frugiperda* larva biocontrol. Finally, these findings offer a scientific basis for future large-scale field trials.

## Data Availability

The raw data supporting the conclusions of this article will be made available by the authors on request.
